# Ovarian Serous Cystadenoma Presents As Bladder Issues in 23-Year-Old Female: A Case Report

**DOI:** 10.7759/cureus.23033

**Published:** 2022-03-10

**Authors:** Yakubmiyer Musheyev, Maria Levada, Benjamin Ilyaev

**Affiliations:** 1 Medicine, New York Institute of Technology College of Osteopathic Medicine (NYIT-COM), Old Westbury, USA; 2 Obstetrics and Gynaecology, New York Institute of Technology College of Osteopathic Medicine (NYIT-COM), Old Westbury, USA; 3 Medicine, Hofstra University, Hempstead, USA

**Keywords:** unable to void, abdominal bloating, urinary retention, ovarian cystectomy, oophorectomy, serous cystadenoma, ovarian cyst

## Abstract

Pelvic organ problem(s) should be suspected when a female patient experiences difficulty emptying her bladder (urinary retention), abdominal distention, and bloating. Clinical suspicion is increased if she also reports any sexual activity while not using barrier contraception or is inconsistent with the use of barrier contraception as this can increase the likelihood of a sexually transmitted disease which can ultimately mimic the same symptoms. Exams that aid in the diagnosis of bladder issues include bladder ultrasound, urine analysis, and cystoscopy. Ovarian serous cystadenomas are common benign epithelial neoplasms that can range in size from 1-30 cm, and can also mimic symptoms/signs associated with bladder issues. In this case study, we present a 23-year-old female patient that presented to the clinic with signs and symptoms of bladder issues including difficulty voiding and abdominal distention. Upon further workup of the patient, it was evident that the patient had a large cyst of the right ovary that was surgically removed. A pathologic exam revealed that it was a benign serous cystadenoma that measured an impressive 28 cms in diameter.

## Introduction

Difficulty voiding (unable to empty one’s bladder) is one of the most prevalent urological complaints resulting in patients presenting to the emergency department and this inability to pass urine can be acute or chronic [1]. The diagnosis of urinary retention is made by filling the bladder then measuring post-void residual volume ultrasound or straight catheterization: results showing greater than fifty milliliters is a sign of retention. Two of the most common causes of chronic urinary retention in women are bladder muscle dysfunction and obstructions [1].

It is interesting to note that in previous literature it has been established that sexually transmitted diseases can cause urethritis leading to urethral edema which ultimately results in urinary retention [2]. Urinary retention can then directly lead to a distended bladder, and complaints of bloating and abdominal distension from a patient.

Benign serous tumors of the ovary represent sixteen to twenty percent of all ovarian epithelial neoplasms and account for two-thirds of benign ovarian epithelial tumors [3]. More specifically, serous cystadenomas do not have mutations in either KRAS or BRAF in contrast to serous borderline tumors and low-grade serous carcinoma, furthermore, mucinous cystadenomas also has a mutation in KRAS [3]. It is interesting to note that cystadenoma is likely to be a characteristic feature of the subgroup of families with hereditary ovarian cancers unassociated with BRCA1/BRCA2 constitutional mutations [4]. This information of genetics is important because it allows physicians to reassure patients (diagnosed with serous cystadenoma) that their condition is not implicated with mutations that could cause other disease states such as colorectal cancer (associated with KRAS) [5] and melanoma (associated with BRAF) [6]. 

Small ovarian cystadenomas ranging in size from 1 to 3 cm are usually incidental findings and reveal themselves during an ultrasound investigation of another gynecologic disorder [7]. The symptoms and signs associated with large tumors are nonspecific and most commonly include mass effect, pelvic pain, bloating, and discomfort [8].

It is possible for patients with ovarian cystadenoma to present with issues that mimic bladder problems. These problems include urinary retention and/or distention.

## Case presentation

A 23-year-old female presented to her Obstetrics and Gynecology (ObGyn) physician with complaints of bloating and being unable to void. Her family history revealed nothing significant while her social history showed that she was sexually active using oral contraceptives and was a non-smoker. Her past medical history was significant for anxiety for which she took Lexapro. Physical exam of the pelvis and genitalia revealed what appeared to be a palpable distended bladder. An ultrasound revealed excess fluid in the region (both ovaries were visualized during this first initial ultrasound and were noted to be normal). A complete blood count (CBC) and urinalysis revealed no abnormalities. The patient was deemed stable, by the physician and was referred to a urologist for further evaluation. The patient was worried because she had never experienced such symptoms before. 

The differential diagnosis for a patient presenting with bloating, inability to void, and a distended bladder with visualized ovaries is urinary retention secondary to obstruction, bladder muscle dysfunction, or cystitis.

**Figure 1:**
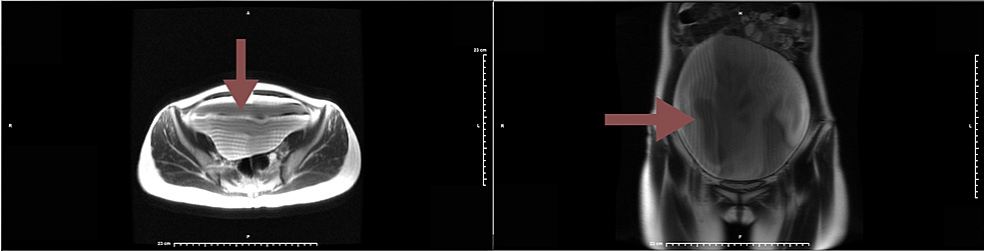
MRI transverse (left) and MRI coronal (right) – midline cystic lesion (red arrow)

Because of the patient's complaints, she was initially diagnosed as having an unspecified bladder disorder. The patient was referred to a urologist but was noncompliant and instead returned to the ObGyn’s office five months later. The patient then underwent another ultrasound of the pelvis (transvaginal and transabdominal) as well as a duplex scan of the arterial inflow and venous outflow of the pelvis. These tests revealed a large right unilocular adnexal simple cyst extending to the midline, distending the anterior abdominal wall, measuring 21.8 x 10.8 x 19 cm. An empty urinary bladder was appreciated at this time. The left ovary was visualized but the right ovary was not. The decision was made to further evaluate these findings with an MRI of the pelvis with, and without contrast. It revealed an extremely large cystic lesion in the abdomen and pelvis (Figure 1) that now measured 24.4 x 23.2 x 2.5 cm. This cyst displaced the surrounding abdominal contents, the wall of this lesion appeared thin and no mural nodularity was appreciated. The bladder wall was mildly prominent which likely reflected underdistention and there was trace free fluid in the pelvis. This lesion could represent a giant mesenteric cyst or possibly an adnexal cyst. The MRI also revealed that there were no further abnormal areas of growth within or near the cyst. Radiology recommended surgical excision if clinically warranted, if not, close follow op was recommended. In a follow-up visit after these scans, a larger mass was palpable 4-6 cm above the umbilicus and the patient was advised that because of the size of the cyst extending above the umbilicus, the procedure for excision would be done via a small low transverse abdominal incision. 

**Figure 2:**
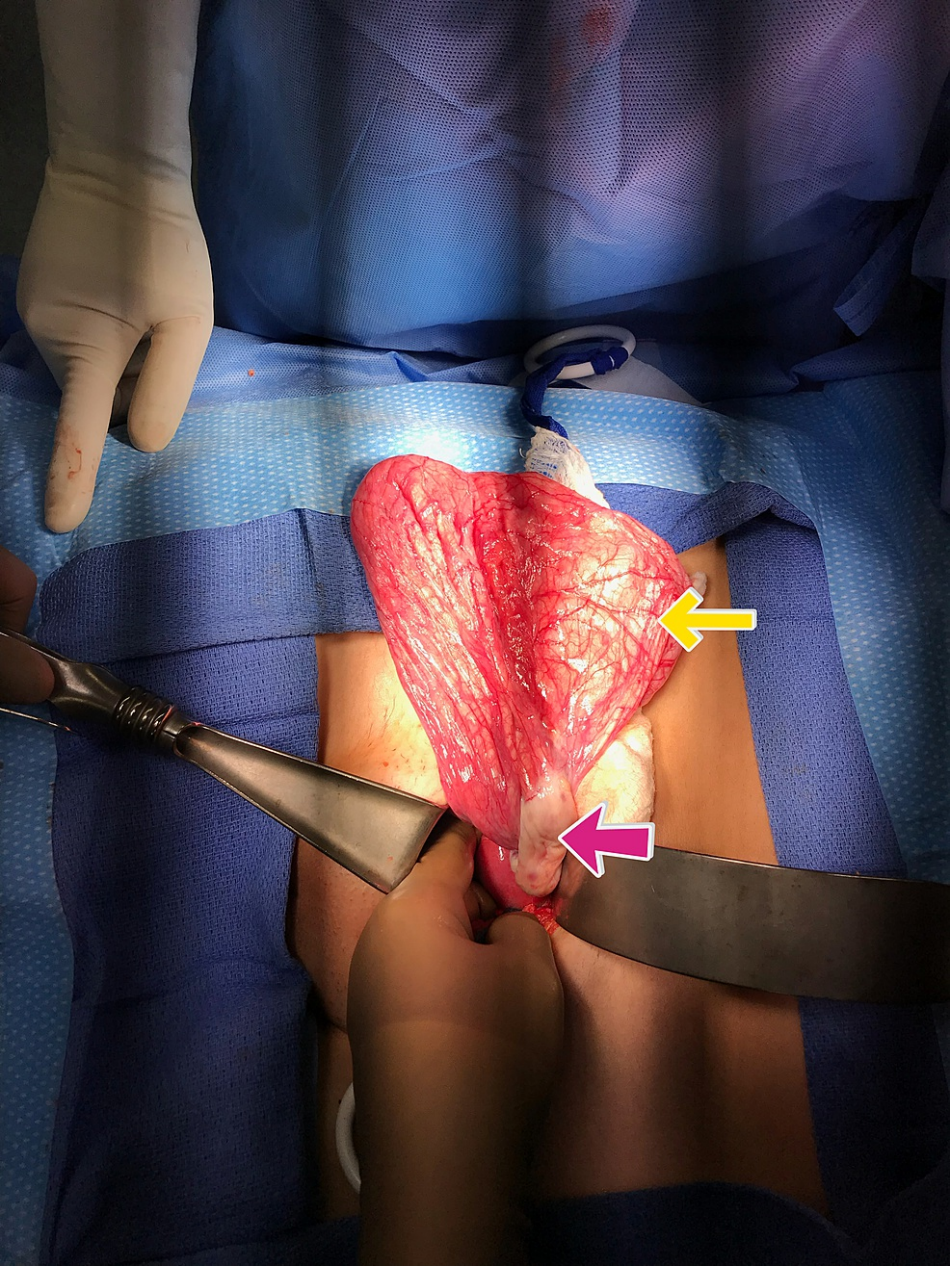
Intraoperative cyst wall externalization(yellow arrow) with view of the right ovary(red arrow)

The patient was scheduled for an ovarian cystectomy with a discussion for a possible oophorectomy. The surgeon, through a low transverse abdominal incision, entered the peritoneum, and obtained washing for cytology: the surgeon then inserted the suction aspirators into the cyst which yielded approximately three liters of clear fluid(from a 26-28 cm cyst) which was sent to cytology for analysis. The cyst wall was then completely dissected away from the remaining (normal) ovarian tissue (Figure 2) and sent, in its entirety, to pathology. The right ovary was able to be visualized and saved, and oophorectomy was not performed. The preoperative diagnosis was a complex cyst of the right ovary. The postoperative diagnosis from pathology was benign serous cystadenoma. The cytology reports showed that there were no malignant cells present. It is interesting to note that pathological and cytological testing was not performed intraoperatively and was performed after the surgery in order to garner a final conclusive diagnosis.

**Figure 3:**
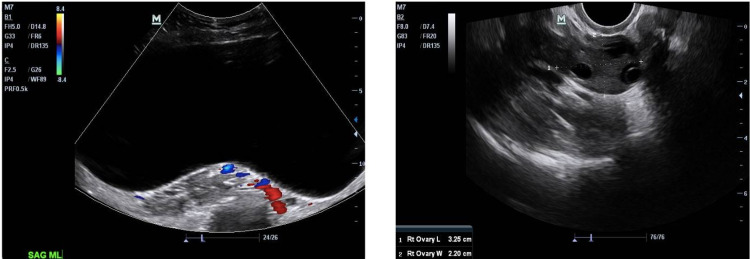
Preoperative (left-benign serous cystadenoma) and postoperative ultrasound (right-normal) images of the patient.

Initial therapy for a complex cyst of the right ovary is surgical removal. The patient was discharged on the third postoperative day with pain medication for the first week and followed up in the office on post-op days six and 21. On both appointments, the patient's incision was observed to be healing well and was clean, dry, intact, and non-tender. After discussion, the patient and physician decided to conduct another ultrasound in order to confirm that everything was normal and reassure the patient. This postoperative ultrasound was completed and confirmed there were no abnormalities to the abdominal region and the ovaries were able to be appreciated. This is in contrast to the preoperative ultrasound which showed excess fluid that did not allow much of anything else to be visualized (Figure 3).

## Discussion

The differential diagnosis for a patient presenting with complaints of difficulty urinating and bloating is vast. The history and physical examination of the patient should be centered around the urinary symptoms involving the lower genitourinary (GU) tract: the symptoms include but are not limited to blood in the urine, lower stomach pain, discomfort with the evacuation of urine, foul-smelling urine, or urethral discharge [1] as well as not being able to empty the bladder. The physician should also make it a point to ask about operations, previous trauma, or the use of radiation to the lower genitourinary area as well as inquire if there is a past medical history of pyrexia, lumbar/sacral pain, neurological symptoms(such as perineal numbness) or intravenous drug administration [1]. All kinds of supplements and medications, that involve prescription, herbal, and over-the-counter (OTC), should be assessed in order to find out if these medications have an undue influence in causing urinary retention [1].

Regardless of whether the patient's history is pertinent, imaging studies and a urine analysis must be ordered. The first scan ordered could be an ultrasound and depending on the results, further scans may be warranted. 

It is interesting to note that even though an MRI scan can reveal useful information, it is still very difficult to establish a specific diagnosis based solely on imaging. To clarify, in this case, the preoperative diagnosis was a complex cyst of the right ovary. It was only after surgery that the physician was able to definitively diagnose the patient with benign serous cystadenoma. We see here that the diagnosis of ovarian cystadenomas is definitively based on the histopathological report garnered after surgical excision of the cystadenomas; this being said, the cell type of cystadenomas cannot adequately be determined solely according to imaging findings [3].

Curiously enough, the treatment and care of ovarian cystadenomas are largely based upon the symptoms experienced by the patient. Other factors at play include the age(and menopausal state of the patient), size of the cyst, and medical history: the main treatment modality of ovarian cystadenomas is unilateral salpingo-oophorectomy or ovarian cystectomy [3]. After surgical excision, clinical recurrence is rare and usually signifies that there is a new primary neoplasm at hand or the tumor was incompletely resected [3].

As we see in this case, it is possible for patients with ovarian cystadenoma to present with issues that mimic bladder problems. This is why it is important for any clinician to keep an open mind when seeing female patients who present with genitourinary complaints. It is also important to note that the serous cystadenomas can range in many sizes: they can span from 1 to more than 30 cm in the greatest dimension (mean = 10 cm) [3]. Because of this, our patient’s serous cystadenoma size is of the utmost interest.

## Conclusions

Ultimately, in this report, we present some signs, symptoms, and laboratory examinations used to evaluate bladder issues such as urinary retention and obstruction. Furthermore, we elaborate on the signs, and symptoms that are used to diagnose ovarian cysts and how these results may overlap with those found with bladder issues. This possible overlap was evident in this case study in which a female patient presented with what appeared to be signs of bladder issues but ultimately ended up being a large ovarian cyst which was surgically removed and diagnosed by pathology to be a benign serous cystadenoma. 

To reiterate, the main takeaway from this report is that it is integral for medical professionals to always have a broad differential when taking care of female patients who complain of genitourinary complaints. Furthermore, as previously mentioned, serous cystadenomas can reach sizes up to 30 cm. Because of this, our patient’s serous cystadenoma size of 28 cm is of the utmost significance.
